# Ethics and diversity in artificial intelligence policies, strategies and initiatives

**DOI:** 10.1007/s43681-022-00218-9

**Published:** 2022-10-06

**Authors:** Cathy Roche, P. J. Wall, Dave Lewis

**Affiliations:** 1 Science Foundation Ireland, CRT-AI, Trinity College Dublin, Dublin, Ireland; 2 ADAPT Centre, Trinity College Dublin, Dublin, Ireland

**Keywords:** Artificial intelligence, AI ethics, Diversity, Intersectionality, Power

## Abstract

A burgeoning of Artificial Intelligence (AI) technologies in recent years has led to increased discussion about its potential to address many issues considered otherwise intractable, including those highlighted by the United Nations 2030 Agenda for Sustainable Development and associated Sustainable Development Goals. In tandem with this growth in AI is an expanding body of documentation regarding how such advanced technologies should be governed and managed. Issued by a variety of sources and comprising frameworks, policies and guidelines, this body of work encompasses the legal, social, ethical and policy issues around AI. With at least 470 such documents identified, as of May 2021, in the Council of Europe’s tracker of AI initiatives, questions are emerging around the diversity of views expressed, especially regarding the influence of the Global North or Euro-American perspectives. Our previous analysis of a corpus of largely grey literature discovered blind spots regarding both gender representation and perspectives from the Global South. Expanding on that work, this paper examines a significantly extended corpus, with a focus on the role of underrepresented groups in the wider AI discourse. We find that voices from the Global South and consideration of alternative ethical approaches are largely absent from the conversation. In light of the prominence of social, cultural and ethical perspectives from the Global North, this paper explores implications for the development of standards for ethical AI. Concluding by offering approaches to incorporate more diverse ethical viewpoints and beliefs, we call for increased consideration of power structures when developing AI ethics policies and standards within these alternative socio-cultural and socio-economic contexts.

## Introduction

The rapid advancement of AI technologies has stimulated vigorous discussion about their transformative potential, and the manner in which these technologies can reconfigure work and personal life. Although the primary motivation driving the use of such advanced technologies may focus on the economic benefits arising, it is clear that AI and related technologies have the potential to address problems in sectors as diverse as business management, agriculture, education and healthcare [1–4]. AI is even promoted as a mechanism to address challenges previously considered intractable, such as sustainable development, including the Sustainable Development Goals (SDGs) [5], with a recent study claiming that AI could enable the accomplishment of 134 of the 169 agreed targets across all goals, highlighting the potential “*to unlock benefits that could go far beyond the SDGs*” [6, p. 8].

In tandem with this awareness of the potential benefits of AI, policymakers and researchers have identified problems across a broad range of applications. These include such issues as semantic biases in Machine Learning (ML) [7]; machine ethics and cybersecurity for autonomous vehicles [8]; gender bias [9, 10] and the role of AI in enabling misinformation and disinformation across social media and other platforms [11, 12]. Evidently, real-world applications of AI technology often have unanticipated impacts and outcomes, leading to debate about how such advanced technologies should be designed, managed and governed. Efforts to address these risks and the many other social, cultural and ethical implications of AI have resulted in an ever-growing body of documentation seeking to create and establish AI policies, frameworks and guidelines. Issued by a variety of international agencies, non-governmental organisations (NGOs), governments and the private sector, these principles, declarations and strategies have attracted widespread attention as they are likely to determine how both public and private bodies will design, develop, implement and regulate AI into the future.

Of importance, is the claim that the research and production of policies around the ethical dimensions of AI have been dominated by countries in Europe and North America, who are over-represented in terms of the production of AI ethics policies and frameworks [13, 14]. Perhaps this is not surprising, given the prominence of these countries in the development of such advanced technologies and the greater availability of resources for policy work in the Global North. Nonetheless, it is problematic, as just like the AI technology itself, the ethical frameworks and standards emerging around it are reflective of the value systems and power structures within the societies in which they are developed. As these Global North values are not necessarily universal and they can and do differ from other cultural groups, any guidelines or policies based on such ethical values may conflict with, and disadvantage, other religions or cultural value systems. Moreover, inherent in such ethical standpoints are matters around the continuation of power and privilege structures, the potential echoing of colonial histories and imbalanced representation in decision-making. It is important, therefore, to understand the nature of the emerging ethical frameworks and standards for AI from the perspective of global participation and to question any perceived or real dominance of voices from the Global North in the international policy and governance discourse.

Of key interest is how to ensure such emerging frameworks and standards themselves do not also reproduce and reinforce a variety of biases or augment inequality through their design, development and implementation. In this regard, the inclusion of a wide diversity of voices in AI ethics policy and debate is especially important. For some commentators, this lack of diversity and the almost ubiquity of Global North influences on the design of AI technologies and algorithms, which involves encoding an understanding of the world through these particular socio-cultural lenses, has resulted in a homogenisation of algorithmic design that philosophically and economically finds itself at odds with cultural philosophies and interests of the Global South [15]. This can be extended to the AI governance policies and approaches currently emerging, which also predominantly encode a Global North perspective on ethics, values and normative understanding. If AI is deployed and governed in this manner and if ethical frameworks do not align with local ethical viewpoints, then the desired trustworthiness of the AI solution may be difficult to achieve, uptake impaired and, therefore, its potential global benefits are unlikely to be realised.

Given this context, this paper asks whether ethical considerations around AI are inclusive, specifically in terms of reflecting and representing the interests of those in the Global South. While recognising that technological development inevitably outpaces governance and regulatory policy, we ask whether ethical approaches can be established to support AI technologies and their governance that are reflective of multiple national, social, cultural and ethical contexts? The objective is to establish a path towards ensuring that future AI ethics frameworks and guidelines reflect the diverse communities they purport to serve.

While progress has been made in the twenty-first century to reduce inequalities, inequity still persists, with over 700 million people or 10% of the world’s population living in extreme poverty [16]. Furthermore, inequality across “*a wide range of enhanced capabilities*” [17, p. 10], including those relating to advanced technology, is rising, with the gap between high and low human development countries widening in terms of digital technology access such as mobile telephony and broadband. While technology and AI can bridge the divide between countries in the Global North and the Global South, it can also generate various gaps between them. As the body of AI policies and studies continues to expand, it is appropriate to examine and analyse this literature from the perspective of diversity and inclusion. As AI is being implemented globally, discussions on ethical frameworks that ignore the context of their application, such as culture, socio-economic, environment and gender, can inhibit the achievement of all SDGs. Moreover, if the discussion around ethical questions raised by AI is occurring largely in the Global North, it is possible that any identified solutions might not be suitable for underrepresented populations, both within the Global North and the Global South. Issues affecting such populations may be ignored completely or not given adequate consideration within a literature dominated by the Global North and efforts to shed light on the inclusivity of the AI documentation will help identify ways to address inequalities or at the very least, not amplify them. An initial analysis by the authors of an independently collated corpus of AI ethics documents (*N* = 84) identifies two particular blind spots in the emerging policy and regulatory guidelines around AI: namely, voices from the Global South and those of women more broadly [18]. This paper significantly expands our previous work, examining a greater body of AI policies, studies, and governance frameworks in greater depth. In addition, this paper examines the extended body of AI documents with a lens on socio-cultural diversity and socio-economic disparity by focussing on underrepresented groups, especially relating to the Global South.

We have carefully considered the terminology used in this paper, as there are a wide variety of terms used to refer to countries and underrepresented populations in the literature and documents referenced by this research. These include Global North, Global South, developed, underdeveloped, first-world, second-world, third-world, the West, the East, the South, the BRICs (Brazil, Russia, India, and China), low- and middle-income countries (LMICs), high-income countries, resource-constrained and high-resource countries. Although the subject of much debate, there is still little consensus about which is most appropriate in any given context. Walsham refers to “*so-called developing countries*” [19, p. 2], whilst Heeks notes that “*some people don’t like the term (developing country): the idea that countries like the US and UK are 'developed' is clearly ridiculous if we equate this with them being the finished article*” [20, p. 10]. Although it is recognised that Global South and Global North are not entirely unproblematic, these have been chosen for this research as the term Global South functions as more than a metaphor for underdevelopment [21]. In using these terms, this paper also recognises that the Global South is not a single entity, with much variety both between and within countries.

Seeking to identify and address inequity across contexts in AI ethics discussions, specifically between the Global North and Global South, this work is positioned within the fields of information systems and ethics, and specifically within the sub-fields of AI ethics for global development (AIethics4D) and Information and Communications Technology for global Development (ICT4D). Additionally, the work will be of interest to those working with intersectionality and gender studies. This paper should therefore be of interest to both academics and practitioners working within these, and other, related fields. An important contribution to a diversity of perspectives on AI ethics, this research provides evidence-based substantiation of an approach to AI ethics that is reflective of predominantly Global North perspectives and concerns which, if unchecked, could further exclude underrepresented groups. The paper proceeds as follows: firstly, we examine the literature on AI and the related areas of power structures, ethnocentricity, intersectionality and inclusion. Proceeding with a description of the research methodology adopted in Sect. 3, before presenting findings and their associated discussion in Sects.4 and 5, respectively. The paper closes with our conclusions and a call to action in Sect. 6.

## Literature review

The body of work on AI is expanding rapidly, not just regarding AI policy but also around ethical guidelines and principles and the formulation of binding and non-binding instruments for AI. This section commences with a brief overview of the AI literature, before exploring work on social and power structures in AI. An overview of ethnocentricity and ethics is offered before discussing recent trends in the literature, including data feminism and data justice. The section concludes with a discussion of a potential theoretical paradigm to address the issues identified, namely intersectionality.

### AI policies and strategies

Accompanying the rapid growth of AI and other such advanced technologies is a flourishing of documents on all aspects of AI. Of particular interest here are those concerned with providing principles, frameworks and otherwise defining policy in terms of potentially normative guidance. Several studies have pointed to convergence around what ethical principles should be considered when developing or implementing AI. One such study of 36 AI principles documents identified eight common themes, including privacy, accountability, transparency and promotion of human values [13]. Another study of 84 so-called ‘soft-law’ documents or grey literature also found consensus around similar principles of fairness and justice, transparency, privacy and responsibility [14]. While focussed more specifically on the ethics of AI in the business domain, a study of 47 AI ethics guidelines (combined from three existing studies), perhaps unsurprisingly, identified fairness and accountability as the two most widely observed principles in AI business practices [22]. Furthermore, a recent analysis of 200 AI ethics guidelines and recommendations, which, while more geographically spread than in previous surveys, still identifies a focus on the five most common principles remarkably similar to other studies, with a focus on transparency, reliability, accountability, privacy and fairness [23]. Lamenting the “*apparent unwavering distribution of documents into world regions/countries*” [23, p. 15] the authors find that the majority of the documents analysed come from Europe, North America and Asia, with regions such as South America, Africa and Oceania representing less than 5% of the entire sample size.

What is clear from the range of survey studies reviewed, is that, regardless of domain or subject matter being addressed, thematic trends have and are emerging across a range of AI ethics guidelines. Pointing to a broad level of incipient consistency around what ethical principles should be prominent in the AI discussion, the literature offers “*a deterministic vision of AI/ML, the ethics of which are best addressed through certain kinds of technical and design expertise*” [24, p. 2129]. Perhaps reflective of the role of large AI companies in the production of guidelines and value statements around the ethics of AI, there is no real suggestion of any constraints or limitations around AI. Furthermore, the literature also shows that this emergent consensus is based on documents largely from the Global North.

However, there is also some divergence emerging across the body of policy documentation, particularly in relation to the provenance of the documents. Examining 112 documents for reference to 25 identified ethical topics, it is posited that there are clear differences in ethical breadth between documents from private entities and those issued by non-governmental organisations or the public sector [25]. Furthermore, despite also identifying recurring topics common to those found in previous analyses of guidelines and principles, the value of such agreement on ethical themes can be called into question as there can be a profound gap between theory and practice. As noted by Hagendorff, given that AI “*lacks mechanisms to reinforce its own normative claims*” [26, p. 99], broad agreement on the ethical principles of AI can have little meaning in practice whereby deviations from the code of ethics have few consequences. Nonetheless, the AI literature is indicating areas of focus, convergence and divergence on AI ethics which, while still largely discursive in nature, have the potential to be implemented tangibly as directions for action. Furthermore, the emergence of such studies based on the collection, cataloguing and sharing of large samples of AI literature demonstrates how specific questions around identified foci within AI ethics can be investigated more systematically than reliance on specific use case studies permits. This paper embraces and contributes to this breadth-wise empirical turn in global AI policy research. Having identified from the literature a concern with a limited set of principles, and a focus on technical expertise embedded in a principally Global North context, implications of this existing approach to AI ethics and some alternative conceptualisations of ethical AI, are explored in the following sections.

### Social and power structures

Whether or not such codes are enforceable or binding, the ethical principles and values being proposed mirror and replicate the context in which they are developed. Crawford argues that while AI is often presented as “*disembodied intelligence, removed from any relation to the material world*” [27, p. 7], and therefore as neutral and objective, it is not incorporeal and because it is subject to human ideologies and viewpoints, is necessarily prone to bias. Moreover, a systematic analysis of the values encoded in ML research revealed the discipline itself to be inherently value-laden, moreover, it is considered to be *“socially and politically loaded, frequently neglecting societal needs and harms, while prioritizing and promoting the concentration of resources, tools, knowledge, and power in the hands of already powerful actors*” [28, p. 182]. As a constructed entity, intelligence cannot be removed from the influence of social, political and cultural influences in which it is created. To argue otherwise, enables a separation of AI from its various impacts, including the socio-cultural and environmental effects, facilitating a dismissal of bias as simply a technical issue. To understand AI and its driving forces, it is necessary to situate the technology within the power relations reproducing it.

Acknowledging that technology is heavily influenced by those *“who build it and the data that feeds it”* [29, p. 1], it, therefore, reflects the power and social structures of its context. It is clear that the impact of AI is not felt equally and that these technologies can potentially further the divide in global digital inequality. This is especially true amongst often marginalised populations, such as those with disabilities, ethnic and racial groups, LGBTQ + and young people, those in poor communities, both rural and urban, women and all those at the intersection of such identities. One example, often cited because of its sheer prevalence, is that of gender bias reproduced within and by AI systems [9, 10, 30, 31]. This bias has very real and potentially devastating consequences as noted by a recent United Nations Educational, Scientific and Cultural Organisation (UNESCO) report, which states that “*these gender biases risk further stigmatizing and marginalizing women on a global scale*” [32, p. 4] and that due to the ubiquity of AI, such biases increase the risk of women being left behind in all aspects of life. Whether on the basis of gender, or inequality on any other grounds, there is incontrovertible evidence that AI replicates the injustices of the society in which it is created.

If computing systems are “*proxies for the people who made them*” [33, p. 67], the historic lack of diversity among those who design such systems means that the beliefs embedded in the technology are reflective of a narrow, non-diverse perspective. Similarly, the documentation around the purpose, governance, direction and development of technology can be seen as representative of its social, cultural and ethical context. As Roff observes, as humans, we live *“in a complex web of social interactions, norms, customs, and power relations”* [34, p. 135]. Within this ‘web’, exist social realities which are constructed and changeable and therefore cannot be universal. As a logical extension, any proposed AI ethics and associated values reflect the constructed nature of reality and cannot be assumed to be applicable in all contexts, especially in those environments with very different power structures and diverse cultural, social and ethical outlooks. Correspondingly, there is “*no universal consensus on which positions to take*” [35, p. 8], and while one can agree or disagree with a particular ethical stance, this too requires adopting a position on the underlying principles. This has implications for AI’s impact on such things as human rights and will raise questions that will “*take on ever greater relevance over the next few decades*” [36, p. 155]. The key point here is that AI policy documents and ethical frameworks are not neutral but embody a certain philosophical and cultural world view.

### Ethics and ethnocentricity

As evident from the overview of the AI documentation, consensus is emerging on the central ethical themes of AI, such as privacy and transparency. Additionally, from the discussion on social and power structures, it is apparent that such convergence indicates these ethical values are the product of a shared prevailing culture. Within this environment, there is a risk of ethnocentrism, whereby other cultures are evaluated through the standards and perspectives of the prevailing culture. Such an approach has especial significance when talking of AI and the Global South, with historically unequal, colonial and biased social and power structures.

Digital, technological, electronic and data colonialism, technological imperialism, algorithmic coloniality are all terms used to refer to exploitation and dispossession of the Global South in the emerging technological, data and AI-driven order. While a variety of definitions abound, all share a focus on the central issue of the extractive and exploitative nature of AI in these contexts. Concern about US infrastructural dominance of the digital ecosystem in the Global South has led some authors to identify an insidious “*digital colonialism*” [37, p. 4] at play, shaping the digital future of many African nations. Additionally, in their study of how low or middle-income countries are made legible in the development context, Taylor and Broeders discuss power dynamics amongst development actors and how power has shifted from the state as collector and user of statistics to a more distributed model of governance, wherein power lies with those who hold the most data. In many cases, these are corporations and therefore have become development actors in a sort of “*informational capitalism*” [38, p. 229]. Furthermore, a burgeoning of AI applications as the solution to social problems can lead to “*algorithmic colonialism*”, whereby ideological, political and economic dominance is achieved through a focus on “*state-of the-art algorithms*” and “*technological innovation*” [39, p. 391]. With such perceptions of technological imperialism, it is vital that the ethical guidance and governance around AI do not reinforce such fears or augment existing inequalities by embedding philosophies and ideologies originating in the Global North.

While it can be argued that the phenomenon of data colonialism affects all globally, in that data is extracted and commodified all over the world, the power relations at play in the process are not equitably structured. Data colonialism can be characterised as an “*emerging order for appropriating and extracting social resources for profit through data, practiced *via* data relations*” [40, p. 8], and this colonialism is taking place in the setting of the interlocked history of colonialism and capitalism. Emerging power structures can be seen to have at least two axes of power, that of the Global North and China. In this data-driven world, new types of corporate power have emerged, whereby human life is appropriated and extracted, in the form of data and used to generate profit. Indeed, the resultant power of certain technology companies is now beyond that of some states. That imbalance of power relations and dynamics is omnipresent, although not necessarily manifest, in our daily lives. For example, in their tracing of the life cycle of a single Amazon Echo (voice-enabled AI system), Crawford and Joler [41], document their difficulty in tracking the source of its components, to study how user data is harvested and processed, to track disposal in countries like Pakistan and Ghana: the full scale of extractive nature of AI is difficult to quantify as it involves hardware and infrastructure but also energy consumption of large models and labour exploitation through digital piecework. It is an ever-repeating cycle, prompted by the asking of a question, in which the user becomes part of the product by helping to train the neural networks driving the AI device, leading to the creation of new accumulations of wealth and power, “*concentrated in a very thin social layer*” [41, p. 9]. At play in this new social order are inequities of access, of wealth and of power, similar to the extractive industrial colonialism of previous centuries.

Consequently, recognition and awareness of coloniality, which is “*what survives colonialism*” [42, p. 5], and seeks to explain the power dynamics between coloniser and colonised, that continue after colonialism, should be fundamental in thinking around AI ethics. One approach is that posited by proponents of decolonial AI, whereby acknowledgment that colonial continuities persist should inform considerations of inequality and power regarding AI and therefore must take account of this history, especially in the context of the emergent power of AI corporations. Underpinned by a belief in decoloniality as an appeal to both critique and strive to undo, the logics of coloniality and race that are still operational in AI, Adams warns against the sublimation of decoloniality “*as another rationality that justifies and legitimates AI*” [43, p. 190]. Rather, decolonialising AI demands an appraisal of and challenge to, how AI is made possible by and depends upon, colonial power structures and the dividing practices of racialisation. Similarly, in analysing the emergence of a ‘decolonial turn’ in technology and data studies, Couldry and Mejias also make the case for explicit acknowledgment and exposure of the colonial heart of current data and technology practices [44]. If colonial processes are at the core of AI and are being intensified by the vast expansion of these technologies, a decolonial (albeit a contested term) perspective, is essential when discussing the ethics around the development and use of AI. For Mhlambi and others, this starts with challenging the very language used to talk about AI, a language that is predominantly Western, male, white and wealthy [45]. In their AI Decolonial Manyfesto,^1^ the Western-normative language of “ethical” AI is rejected if it fails to challenge and address power asymmetries.

Speaking of the marginalisation of non-Western knowledge systems within AI ethics studies, Segun calls for ways to end this “*epistemic injustice*” [46, p. 99], whereby only Western ideas, problems and solutions are presented. For some commentators, however, neither Western recognition of “*non-Western elites’ contributions to ideas”* [47, p. 242], nor being more accommodating of other cultures is adequate to challenge the production and reproduction of inequality. However, some alternative approaches to decolonising ethics and challenging the dominance of Euromodern philosophical thought or paradigms have been proposed. For example, Hutchings posits the concept of “*pluriversality*” as a “*distinct pathway*” to decolonialising global ethics [48, p. 124]. Calling into question epistemic and technocratic models of ethics, pluriversality goes beyond a simple ontological pluralism, which in itself opens the debate to a diversity of voices but does not go far enough to counter the tendency to separate formulating ethical positions from their application and outcomes. Instead, pluriversality offers the potential to move beyond this limitation and focus *“our attention on what it means to live with others without subsuming them into one world or another”* [48, p. 124].

In tandem with these views are efforts to make explicit the application of various alternatives to Western ethical ideas of AI. Some such approaches include the incorporation of Ubuntu as an ethical and human rights framework for AI governance [49, 50]; an Islamic virtue-based framework, underpinned by the context of Islamic objectives (*maqāṣid*) [51]; bringing a Buddhist viewpoint and the concept of ethical perfection to AI [52] and applying the indigenous idea of sumac kawsay (Buen Vivir in Spanish) to reinterpret global governance [53]. As challenges or alternatives to Global North and Western ethnocentrism, these approaches also highlight how sociocultural values and norms inform the definition and implementation of AI ethics principles. For example, an analysis of the national AI ethics guidelines for South Korea revealed how public values highlighted by the guidelines are reflective of prevalent Korean sociocultural norms, demonstrating a blend of “*instrumentalist norms, Confucian ethics, multistakeholder deliberation, and public value coproduction”* [54, p. 273].

Overcoming ethnocentrism in the discussion of AI ethics clearly involves more than a simple acknowledgement of diverse values and epistemologies. Understanding and accepting that “*Western values are still not universal*” [55, p. 1] is a vital first step, however. Applications of AI and the ethics around these technologies are not confined only to Western societies or the Global North but rather will be and indeed are already being adopted on a global scale. Therefore, cognisance must be taken of a wider understanding of ethics which takes into account not only other contexts and value systems but also comprehends the historical power structures that persist after territorial colonialism.

Related to the issue of digital and data colonialism previously discussed is that of the broader power asymmetries whereby, within technology generally but especially around AI, power is concentrated in an extraordinarily small group. Posing a challenge, not just to achieving inclusive AI ethics but to existing governance frameworks, the basis of the power of these dominant players is rooted in their possession of enormous datasets and control of access to great volumes of data. Not only is access to the datasets protected by law but control is further strengthened by the “*data holders’ peculiar market positions and the presence of entry barriers*” [56, p. 8]. Moreover, the nature of the data is no longer that of single users only but has scaled to processing data of communities, large groups and even countries. This concentration of power and resultant power imbalance has led to a call to embrace a more holistic approach to AI, one that tackles the lack of a framework that can wholly address the societal issues AI has raised. Acknowledging that a legal approach is inadequate in and of itself, Mantelero posits an enriched Human Rights Impact Assessment that is considerate of these ethical and societal issues: a Human Rights, Ethical and Social Impact Assessment (HRESIA). Blending human rights with the local component of societal values, such an approach presents a conceptual framework for thinking about AI that moves beyond the theoretical limitations of the existing legal structure, which was based on data protection law. Understanding that the universality of human rights is itself disputed, the HRESIA model contextualises human rights and socio-ethical values and could be applied in environments where AI regulation is not human rights-based.

In a similar vein, this concentration of power in the hands of the few, has led to AI constitutionalism, which considers AI and big data as fundamental resources in the economy, which, like water and electricity, can be considered essential components of any social and economic development. For some, the value of an AI constitutionalism rooted in societal contexts and cognisant of traditional lines of privilege and power is particularly urgent for communities “*that lie outside of the AI power centres, whose views remain underrepresented in global norm-making and standards-setting*” [57, p. 224], and whose contexts may not be understood by those building and making ethical judgements about the technology. Other approaches to addressing the asymmetrical power dynamics at play draw on contextualised and participative methods to include the values of the communities in which AI solutions are to be implemented, include data justice, intercultural digital ethics and data feminism.

The idea of data justice, as advanced by Taylor, which considers “*fairness in the way people are made visible, represented and treated as a result of their production of digital data*” [58, p. 1], is an evolving determination of ethical paths through a datafied landscape. Arising from a growing sense that discussions around data should engage more explicitly with questions around politics, power and inclusion, data justice also challenges established ideas of governance, trust and ethics [59]. By situating the ethical challenges that datafication presents in a broader social justice context, data justice moves ethical concerns from the realm of data protection and a focus on individual rights to the interrogation of societal and socio-technical factors. Advancing the concept in research and practice in the decolonial context requires re-envisioning data innovation ecosystems but also resisting the entrenchment of “*existing geopolitical and socioeconomic power dynamics”* [60, p. 14], which strengthen Western cultural hegemony. A further challenge has been amplified by the prominence of technology around COVID-19, with technology firms involved in public service provision across education, health, security, transport and border control domains. In this way, asymmetries of power are compounded: not only do technology firms wield control over infrastructures and data, they now have power over technology structures underpinning most basic public goods, with attendant problems for accountability and public good [61]. Centring on equity, recognising and representing plural interests, data justice can be an effective approach to help create and preserve public goods. Indeed, it is at the heart of recent attempts to devise models for sustainable and just data governance [62].

It is worth noting other approaches to addressing the problem of power imbalances through contextualising ethical guidance. Intercultural Digital Ethics (IDE), a sub-field of information ethics and digital ethics research, strives to remedy imbalances by examining the ethical issues around digital technologies from a variety of social and cultural perspectives [63]. Like many of the theoretical and methodological efforts already discussed, IDE faces the difficulty of achieving digital governance frameworks that cater to different cultural ethical values while simultaneously balancing such frameworks on the international stage. To help develop a truly global IDE, Ess posits interpretive *pros hen* ethical pluralism, which retains the integrity of local sources while offering a framework to support a cosmopolitanism that counters “*computer-mediated colonization”* [64, p. 551]. This ethical pluralism and an associated cross-cultural awareness underpin ethical guidelines for internet researchers and point out a way to incorporate cultural differences or be context-oriented whilst embracing a range of ethical frameworks [65]. Similarly, a globally inclusive approach is evident in the UNESCO recommendation on the ethics of AI, which calls for specific attention to be paid to LMICs and similar countries who have “*been underrepresented in the AI ethics debate”* [66, p. 6], with the attendant risks of ignoring local knowledge and value systems and invites research into the applicability of particular ethical frameworks in specific contexts and cultures.

Finally, data feminism has also emerged as a framework for thinking about data and ethics, specifically applied to the field of data science. Drawing on intersectional feminist thought, data feminism is not only about gender but considers the uses and limits of data, in a way that is “*informed by direct experience [and] by a commitment to action*” [67, p. 8]. In some ways, data feminism can be seen as the application of intersectionality to the field of data science. Overlapping with data feminism is the issue of indigenous data sovereignty. Point 30 of the Feminist Data Manifest-No specifically calls out a rejection of “*coercive settler colonial logics of knowledge and information organization*” [68, p. 5], instead committing to tribal nation sovereignties and valuing indigenous data sovereignty.

All of these efforts to contextualise AI ethics can be viewed as responses to the two levels of power asymmetry at play: Western dominance over other value-based or cultural approaches to ethical AI and the associated power of a few, very large technology companies over infrastructure and data. Each approach has merit and each implicitly reminds us of the socio-political nature of AI: from how data is collected and processed, to classification based on socially constructed variables such as gender and race to how and where these tools are deployed. Whether it be Mantelero’s enhanced human rights framework, the social justice sensibility of data justice, the ethical pluralism of IDE or the challenges to binary and hierarchical classification systems offered by data feminism, all advance routes away from a conceptualisation of AI ethics that relies on experts and from viewing bias as something requiring a technical solution. They focus on power and incorporating alternative cultural and value systems into the AI ethics discourse. Related to these methods is the concept of intersectionality, which is the subject of the next section.

### Inclusion and intersectionality

Although there are still calls for “*an inclusive, critical debate about the current dominance of liberal conceptions of ethics in AI*” [69, p. 339], from the previous discussion, it could be countered that such a debate has commenced, albeit it is not yet mainstream. This section considers another alternative conceptual framework to the current hegemony, that of intersectionality, which, emerging from the racialised experience of minority ethnic women in the US, offers a path to examine interdependencies and connections between social systems and categories, by analysing the locus of power. Like other critical theories, intersectionality understands bias as embedded in sociotechnical systems and explains how such systems replicate inequalities while also giving rise to new discriminations. As Lutz observed, intersectionality “*has long left the field of gender studies*” [70, p. 39] and has many applications, including in the fields of education, psychology, sociology, anthropology as well as in law, the political sciences and health. Importantly, applications of intersectionality and other feminist epistemologies have led to both conceptual framings and the development of tools in response to inequality and bias. Intersectionality provides a systemic, sociotechnical understanding of inequity, one that situates knowledge production as a socially and culturally shifting process, involving many human and technological actors [71, 72]. Emphasising that technical tools are neither neutral nor objective, intersectionality positions these tools amidst sociotechnical entanglements whereby practices and knowledge are necessarily partial, embodied, embedded and situated in a larger nexus of power relations [73, 74].

It is important to note that there is no single definition of intersectionality and indeed, how people understand and use intersectionality is currently characterised by “*tremendous heterogeneity*” [75, p. 5]. However, whatever the characterisation, all conceptualisations of intersectionality share a common concern with uncovering how intersecting power relations influence individual experiences in daily life as well as social relations across societies. As an analytic device, intersectionality is a means of understanding the complexity of human experiences by exploring how class, gender, ability, age, sexuality and race, among others, are interrelated and mutually shape one another [75, 76]. Hancock identifies two intellectual projects within intersectionality discourse: an inclusionary project to make all people visible and an analytical project to reshape the margin-to-centre approach, to create a ‘‘*discourse about analytic relationships among categories of difference*’’ [77, p. 32].

In Crenshaw’s [76] terms, intersectionality speaks to the multiple social identities, social forces and ideological instruments through which disadvantage and power are expressed and legitimised. Simply put, intersectionality recognises that people from different backgrounds experience the world differently, be it due to gender, class, race or other forms of identity. Moving beyond its original application in feminist theory, intersectionality allows for the understanding of the matrix of domination and suppression in which lives are lived. Using an intersectional analytical framework, it is possible to understand how aspects of social and political identities combine to create different modes of discrimination and privilege. Such a framework involves dealing with complexity and flux, understanding that people can be simultaneously privileged and oppressed, for example, white privilege can be heightened or reduced by heteronormativity or educational privilege. Understanding that the extent of privilege changes with the situation and that positions of privilege and discrimination are not fixed, enables a deep interpretation of the structures of power [78]. In this way, intersectionality facilitates inclusion and participation by recognising multiple and overlapping aspects of identity within the nexus of structural and societal oppression and privilege.

With some initial success in the application of intersectionality in AI at the implementation level, there is room for optimism of a broader application of the theory and practice. Within the field of machine learning, intersectionality has been used to address intersectional features within the data in a bid to address algorithmic fairness. Such an approach moves the evaluation of fairness away from looking at a single dimension, like race or gender or ethnicity and instead accounts for intersectional groupings to evaluate fairness across all identities [79–81]. At the theoretical level, the critical concepts of intersectionality could be drawn upon to reconceptualise AI ethics. Offering a more nuanced insight into aspects of diversity and making visible the interdependent systems of discrimination, such an approach intrinsically recognises the context of the individual but also the societal narratives in play. Incorporating feminist, queer and critical race theories, intersectionality is a useful framework to analyse biases built into existing AI, but also to “*uncover alternative ethics from its counter-histories*” [82, p. 3]. Intersectional ethics and approaches can help in re-imagining AI, enabling multiple voices to be heard and be valued. A capacity for real-world change through applications of intersectional theory and practice offers a way to advance social justice and elicit social change through increasing awareness of privilege and by encouraging allyship and ally behaviour [83]. Such an approach could be vital in addressing issues of ethnocentrism, the dominance of Euro-American philosophy and the replication of social and power structures within the current AI ethics discussion.

From the analysis of the literature, it would appear that the emergent consensus around AI ethics is highly ethnocentric in nature, emanating from a liberal epistemic tradition, originating in the Global North. As such, it is reflective of asymmetries in power and lacking in consideration of alternative ethics or diverse socio-cultural contexts. What is required is an approach that acknowledges the complex interplay between the values being promoted within AI ethics and their application on a global scale, including in the Global South. While it can be seen as a relatively recent conceptual framework, intersectionality nonetheless allows for an intercultural and therefore more inclusive approach to AI ethics.

## Research methodology

From the above review of the literature which is largely theoretical and qualitative, it is clear that there are imbalances between the Global North and Global South around AI ethics. To establish or confirm if such an imbalance is also reflected in the policy literature, a broader quantitative assessment is required. Investigating if this perceived monocultural dominance of the Global North is evident in the documentation, a robust data collection process was undertaken to create a large corpus of AI policies, reports, guidelines and ethical principles which could then be analysed from a diversity perspective. This methodology is detailed in the following section, which delineates the process of collecting AI documents and building the database to create the corpus for analysis. A description of the resultant data set is offered before explaining the coding and analysis approach adopted. Taking the approach of providing a quantitative filter on the available data, it is hoped the creation and provision of such a tool will assist researchers in analysing documents without the requirement for a team of research assistants. Enabling a semi-automated initial analysis is especially useful in the context of the profusion of AI ethics and policy documents in recent and current production.

### Document collection

This paper leverages an existing database of AI initiatives as maintained by the Council of Europe (CoE).^2^ This collection is one of URLs, rather than that of documents, papers or projects themselves. While the use of this collection extends the original research [18] greatly in terms of number, from an analysis of a corpus of 84 documents to one exceeding 450 (more than a fivefold increase), it also expands significantly the scope of the work. As the first study was based on the corpus as collected by Jobin et al. [14] for their scoping review of existing soft-law or non-legal norms, it did not encompass legal or academic sources, comprising only grey literature documents. Similarly, that corpus contained only documents that made explicit reference to AI in their description or title and were considered to express a normative ethical stance defined as a “*moral preference for a defined course of action*” [14, p. 320]. Furthermore, sources in that corpus were issued in only four languages other than English (French, German, Greek and Italian). While the authors appended a categorisation to the original Jobin et al. [14] collection to explore potential divergences or similarities across issuing agencies, this was done on the basis of only three broad categories of the issuer.

In contrast, the CoE base is classified into ten types (academic paper, meta-analysis, policy paper, binding and non-binding instruments, methodology (audit/impact assessment), research project, parliamentary proceeding, report/study and frameworks/principles/guidelines) and originating from eight categories of the issuer (private sector, national authority, academia, civil society, international organisation, multi-stakeholder, professional association and think-tanks). The collection, therefore, covers a very broad range of initiatives, from a single-page infographic of guidelines to national policy strategies on AI. Moreover, sources (i.e. country or agency of origin) of the documents encompass 55 countries, 15 languages and several international agencies such as the European Union (EU), the UN and the World Economic Forum (WEF). Documents range in date from 2010 to May 2021, with the majority of the initiatives dating from 2018 and 2019. It is worth noting that there are no documents from 2012 or 2013 included in the original corpus.

As there is no official, authoritative database of AI documents, this collection of URLs garnered by the CoE, encompassing the range of sources and types, can be considered as tantamount to an AI corpus. Similarly, adopting the approach of using an independently defined collection helps evade potential selection bias on the part of the authors were the method of collection based solely on a literature search. While the CoE artificial intelligence site is updated on an ongoing basis, a cut-off date of 1st June 2021 was applied, resulting in a collection of potentially 476 documents. To build the database of documents for this study, it was necessary to verify the links given in the CoE collection and screen for candidate documents. Taking place between 2nd June and 9th July 2021, this screening process consisted of manual verification of the URLs, replacing broken links where possible and identifying alternative versions of the document or initiative in question for inclusion if no longer available at a given location. Where links were to news articles, press releases or blogs, that content was downloaded and stored as PDFs. However, where links were to websites with a variety of content, the authors made a judgement regarding how much to save as indicative content regarding an AI initiative, i.e. a full paper or report, if available or descriptions of projects, if not.

Some URLs brought the user to locations where login access or subscriptions were required. In these cases, access was secured so that the corpus could be as complete as possible. While the majority of links were to English documents or sites, the database also contained URLs to content in 14 other languages, including Mandarin Chinese, Indonesian and Slovak. Official translations were sought and used where available and where not, an unofficial translation was included. In a small number of cases in French and Italian, no translation was available and the documents were saved in their original language for analysis. All new links and saved items were noted and the database was updated accordingly. While all efforts were made to maintain the integrity of the content of the collection, due to the necessary deviations where sources were unavailable due to broken links, lack of translated versions and substitutions for superseded materials, the final synthesis of documents is not identical to the original set of URLs.

### Dataset description

Following the extensive screening and document verification process, the volume of the collection was slightly reduced (*N* = 465). After further deduplication, the corpus was finalised (*N* = 463).

Appendix 1 offers a full list of all AI initiatives by country or agency of origin included in the final collection.

**Fig. 1:**
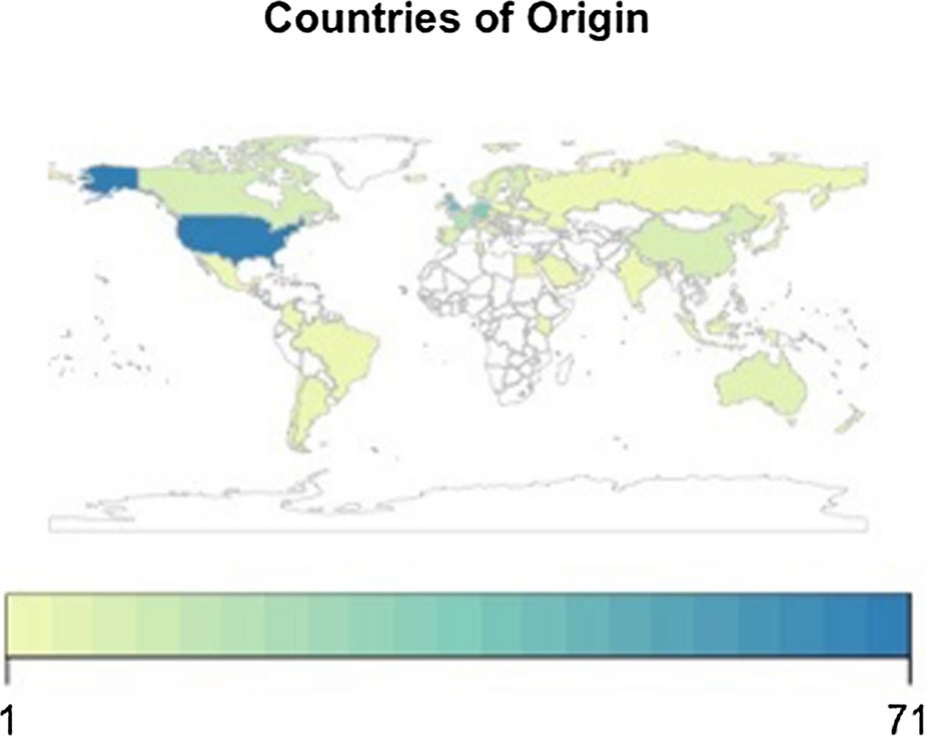
AI initiative by country or agency of origin

Upon initial review and captured in Fig. 1, what emerges is the dominance of a number of countries and agencies within the corpus. The US, United Kingdom (UK) and Germany account for 149 of the 463 documents, representing just over 32% of the corpus. Similarly, a further 124 sources (27%) are contributed by the European Union (EU), CoE and UN. In terms of the document type, three prevailing categories account for a staggering 408 of the 463, or 88% of the collection, namely policy papers, reports/studies and principles/guidelines/charters. Looking at the origin, the majority (289 or over 62%) are from international organisations and national authorities. Tables presenting the corpus by source and document type are available in Appendix 2.

### Content analysis and coding

Coding of the newly created corpus of AI literature to enable analysis for reference to underserved or underrepresented populations was conducted in an iterative manner. Acknowledging that ‘Global South’ is a broad term and subject to interpretation with a specific definition in the context of ICT4D [20], coding was focussed on eliciting the breadth of associated terms to enable comprehensive analysis of the corpus. Therefore, anchor terms encompassed the area of ‘SDGs’/‘Sustainability’ as well as economic conceptualisations of the Global South (e.g. ‘LMICs’, ‘developing countries’) and geographic definitions (e.g. ‘Africa’, ‘Latin America’). While terms such as sustainability and SDGs are applicable to all countries, their origins in Agenda 21 [84] and the Millennium Development Goals (MDGs) [85], with a primary focus on reducing extreme poverty, means they have particular relevance to Global South perspectives. Concerned with sustainable development through poverty eradication, it is likely that AI ethics documents giving prominence to the SDGs can be considered to give some attention to the interests of the Global South. It should be noted that other terms denoting unequal power structures, such as the digital divide and global inequality were not included in this iteration of the research. As the purpose was to maintain a focus on finding a reference to perspectives from the Global South, such terms will be incorporated into future research.

Analysis was then conducted in a phased manner. First, coding for ‘SDGs’ and ‘Global South’ and all related search terms was performed to enable a count per search term within each item in the corpus, as well as overall occurrences of terms. To ensure accuracy throughout the analytical process, manual checks for consistency were conducted by the researchers. This involved regularly checking random samples of the output of the software tool (see a full description of the tool below) to assess its reliability in both finding all occurrences of the defined search terms and not returning terms beyond the scope of this study.

As an iterative process, coding was thereby refined, with additional terms introduced and some removed to ensure the precision and relevance of the search terms. With a starting point of 11 key terms to assess if and to what degree documents in the corpus considered the SDGs or had a focus on the Global South, coding amendments through this process resulted in a large list of related terms. To be as comprehensive as possible in capturing the variety of terms which could relate to such concerns, in addition to existing search phrases, terms such as ‘vulnerable populations’, ‘least developed countries (LDCs)’ and ‘small island developing states (SIDs)’ were added. As in the previous study, some terms deemed unrelated to the theme are excluded, for example, ‘globalisation’ and ‘third countries’. Although not exhaustive, the final collection of terms is complete enough to offer a robust analysis of the corpus. Table 1 presents the final key or ‘anchor’ terms and all associated codes. Codes are not case sensitive and are inclusive of American and English spelling differences.

**Table 1:** Anchor search terms and associated codes

Anchor search term	Included codes
Sustainable	Sustainable, sustainability, sustainably, sustainable development, sustainable society, ecological sustainability, environmental sustainability, sustained participation, agronomic sustainability, unsustainable agriculture, unsustainable, technology [durable, durabilité, sostenibile, sostenibilità]
SDG	SDG, SDGs, Sustainable Development Goal, Sustainable Development Goals, United Nations/UN Sustainable Development Agenda, Millennium Development Goals, MDG, MDGs, Global Goals, [objectifs de développement durable, des ODD, le programme de développement durable, obiettivi di sviluppo sostenibile, OSS, programma di sviluppo sostenible]
Global south	Global South, global justice, global gap, global poverty [sud global, sud del mondo]
Low/middle income	Low income country, low income countries, middle income country, middle income countries, low or middle income country, low and middle income countries, LMIC, LMICs [pays à faible revenu, pays à revenu intermédiaire, paesi a basso reddito, paesi a reddito medio]
Developing world	Developing World, developing countries, developing country, developing nation, developing nations, developing economies, emerging economies, least developed countries, LDCs, small island developing states, SIDs, less affluent countries, vulnerable populations, underrepresented populations, underserved populations [monde en développement, pays en développement, vulnérable, marginalisé, sous-représenté, mondo/paesi in via di sviluppo, vulnerabile, emarginara, sotto rappresentato, il meno]
Low resource	Low resource country, low resource countries, resource constrained country, resource constrained countries, under-resourced states, resource-poor populations [faible ressources, ressources limitées, risorsa/e bassa, risorse limitate]
Africa	Africa, sub-Saharan Africa, African, African ethics (Ubuntu), South Africa [Afrique, Afrique sub-saharienne, Africaine, Africain, Afrique du Sud, Africa, Africano, Africana, Africa sub-sahariana, Sudafrica]
Third world	Third World, third world countries, third world nations [Tiers-Monde, Pays du tiers-monde, terzo mondo, paesi del terzo mondo]
Latin America	Latin America, Central America, South America, LatAm, LATAM, latam, Latina, Latino [Amérique Latine, America Latina]
India	India, Indian [Inde, India, Indiano/a]
Asia Pacific	Asian Pacific, Asia [Asie, Asia, Asiatico]

In the CoE collection, Voyant Tools^3^ a web-based reading and analysis environment for web-based texts is used to calculate a numerical value to indicate the frequency per million of certain concepts within a document. It should be noted that in the CoE set of URLs, this value is only calculated for the category "Principles/Guidelines/Charters", as that is the category considered of most relevance for such an evaluation. In this study, however, the tool is used in a slightly different way due to the differing purpose and is applied to the entire corpus. As a result, the second stage of analysis involved applying Voyant Tools to count both the total number of words (tokens) and the word forms count (distinct words). As the corpus consists of a great variety of document types, there is also a huge variance in length. This step captures the number of occurrences of a search term relative not only to the length of the document but proportional to the ratio of distinct terms and, therefore, overcomes the problem of document size. After calculating the total occurrences of search terms within a document, this was then expressed as a percentage of distinct words, thus preserving proportionality, i.e. a simple numeric value of 20 occurrences does not capture the importance of the terms in a document, as such a number in an item of 200 words is very different to a paper of 10,000 words. Using this percentage, a RAG (Red, Amber, Green) status could be assigned to each document, signalling the level of value of the search terms in each: Red when the theme is absent or very low, Amber when the terms are present but not common and Green when the theme is reflected to a greater extent than in the other two categories.

In the final stage of coding and analysis, results from the first two phases above were used to analyse documents in terms of source (country/agency), origin (issuer) and document type. While the CoE database has since been updated to include a new resource type (website), as this was not a category at the time of analysis, this paper retains the original classification. Although the vast majority of the documents in the corpus are in English (either as a language of origination or translated), five sources in French and one in Italian were analysed in their original language, as translations could not be found and the authors have appropriate proficiency. Codes for these languages are also available in Table 1.

Following the finalisation of the content analysis and coding procedures, data analysis was performed using R in RStudio version 2021.09.0 "Ghost Orchid" Release for MacOS. Much research of this nature is done manually which is time-consuming and subject to human error. Using an automated approach to process the corpus creates potential for this research to be replicable and adaptable to any set of corpora and repeatable over time. Documents were saved as PDFs or converted to the format before applying the ‘pdfsearch’ package in R. Enabling not only keyword searches of PDF files, the package also provides a wrapper (‘keyword_directory’) which loops over all PDFs stored within a directory. Once a character vector of multiple keywords was created, the data was further prepared through various operations such as removing hyphens and ignoring case. As the majority of sources are in the English language (457 of 463), this was set as the corpus language and manual analysis of the five French and one Italian document not available in translation was performed. Outputs were spot checked for accuracy and reliability, as noted earlier.

## Findings

**Fig. 2:**
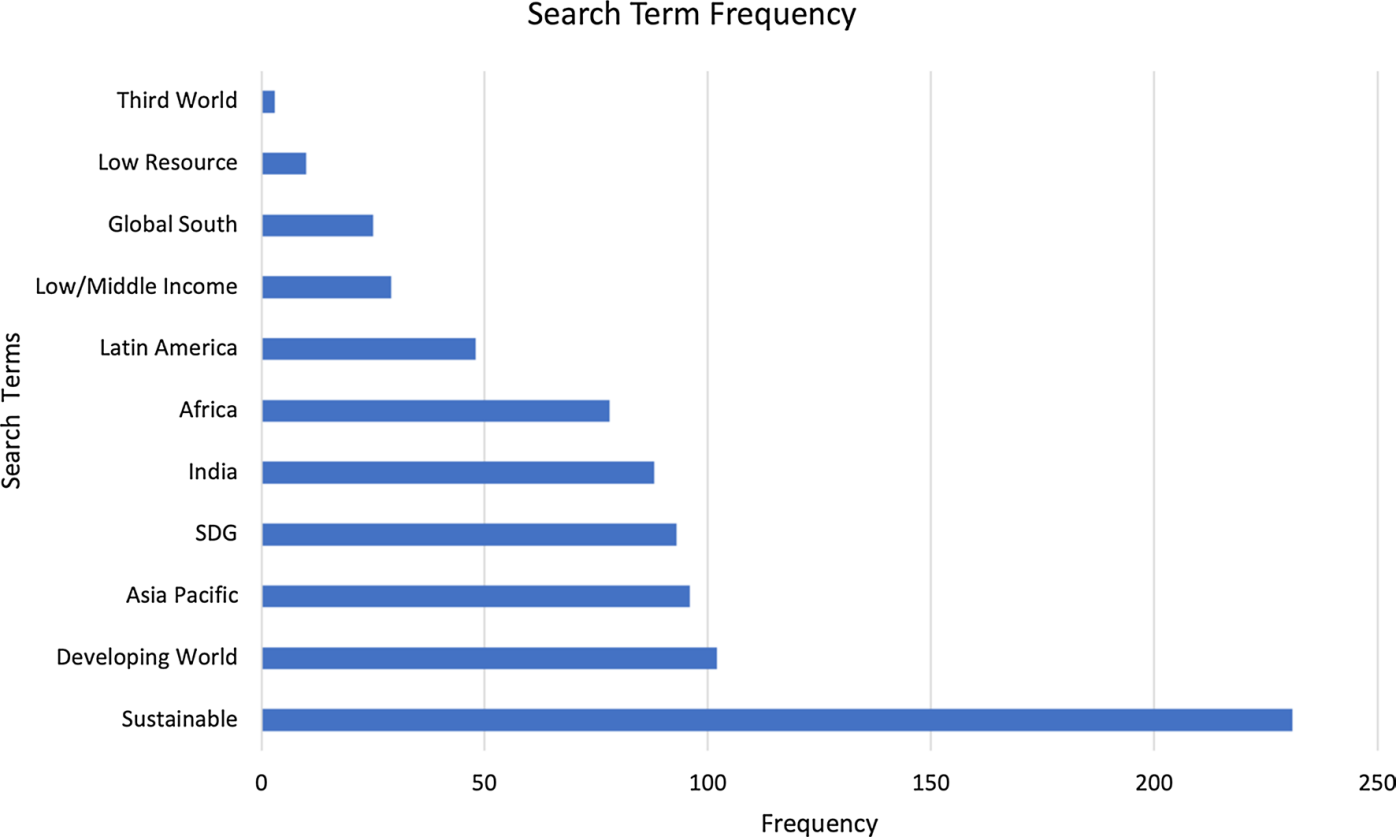
Search term results ordered by occurrence

This section presents the main findings of the research, which may be considered in three dimensions: data sparsity, document origin and document source. As discussed in the previous section, the semi-automated analysis operates as an efficient filter for key terms across this large corpus. What emerges from the findings is a clear overview of which of the search terms are present in the documents and by corollary what is not in the corpus data. Appendix 3 lists the number and percentage of sources in which the key terms occur and the frequency of their occurrence across the corpus is represented by Fig. 2. While an entry was returned for each of the search terms, the sparsity of the data is noticeable, making it almost easier to describe the absence of terms than their presence. For example, while ‘Sustainable’ and associated codes are the most frequently occurring search terms across the documents, the concept is mentioned in just under half of the corpus (49%), meaning the majority of sources do not consider this theme. However, the next most frequently found term, ‘Developing World’, is present in only 22% of the sources. In fact, eight of the 11 anchor terms and related codes occur in fewer than 20% of the documents in this corpus. The least frequently mentioned terms are ‘Third World’, ‘Low Resource’ and ‘Global South’, occurring in 0.6%, 2.1% and 5.4% of sources, respectively.

Excluding references to countries or geographical locations (as very high occurrences in a single document skew the findings), ‘Sustainable’ and associated codes is the term with the highest number of mentions (234) within a document. This is double the frequency of ‘Third World’ which has the second highest number of occurrences (101) in a single source. ‘Low Resource’ and ‘Low/Middle Income’ are mentioned rarely within the sources, with a maximum of two and nine occurrences, respectively, in any one document. Term distribution analysis within the documents reveals again the low level of inclusion of key terms associated with this theme, reinforcing the sparse nature of the data.

The dearth of data points is further evident when the findings are analysed from a whole document perspective (i.e. total occurrences of any or all terms per document), where it emerges that a third (just under 33%) of the corpus have zero references to the theme. Assigning these documents a Red status enabled additional analysis by source, type and origin. Notably, 41% of the sources in the Red category, where all terms are absent, are of the principles/guidelines/charters type while a further 25% are policy papers. Furthermore, 27% of the Red status documents originated with national authorities, 24% came from international organisations and a similar number (22%) from the private sector. Although a variety of countries and agencies are represented in this group, the United States and the Council of Europe account for 38% of all documents in this category. Possible implications of these findings are discussed in the next section.

Looking at the rest of the corpus, where there is some, albeit sparse, occurrence of the key terms and associated codes, the second dimension of findings becomes apparent: that relating to document origin or issuing body. Analysis of the remaining 311 documents, without further subdivision into Amber and Green at this point, reveals that 67% of all sources here have their origins in international organisations and national authorities. Three types of document constitute 90% of this group: namely, report/study; policy paper and principles/guidelines/charters. While again, the documents are sourced from a broad range of agencies and countries, the highest number come from the US, the UK and the EU-European Commission (EC). Separating this group into Amber and Green reveals distinctions in document type but not in origin. When the occurrence of anchor and associated terms are calculated as a percentage of distinct words per document (see Sect. 3.3 for explanation), the maximum percentage is low at 5%. With such a low base, it could be argued that separation into Amber and Green might be a redundant exercise. However, in the interests of potential findings and meaningful analysis, the Amber and Green groups were defined based on between > 1% and < 2% for Amber and > 2% for Green, giving totals of 268 and 43 documents, respectively. With this division, some interesting differences between the two groups are discernible.

A third key dimension of the findings, relating to the source of the document in terms of country or organisation, emerges when analysis is performed across the sub-corpora. Within the Amber subsection of the corpus, the dominance of the US and UK as the sources of the documents is reflected, closely followed by the EU-EC. Policy papers and reports/studies are the main types of sources in this category, accounting for almost 70% of all documents. In contrast to these findings, the Green grouping of sources is led by the UN, specifically UNESCO, followed by the US and Germany. Interestingly, despite their numeric strength in the corpus overall, there are no UK documents present in the Green sub-corpus. Furthermore, the primary types of documents in the Green category are classified as report/study and then principles/guidelines/charters. Absent from this group are academic papers, binding instruments, methodology (audit) and parliamentary proceedings. Although both Amber and Green sets of documents originate primarily from international organisations and national authorities, a subtle difference in the year of issue is apparent, with no documents pre-2014 in the Amber group and none pre-2016 in the Green grouping. Given that the date range for the Red subgroup is 2010–2021, this could be suggestive of a change in focus over time, with more attention being paid to underrepresented groups and global interests in recent years. Again, suggested implications will be discussed in the following section.

Whereas the original content analysis and coding plan included analysing the Green sub-corpus from a qualitative perspective to identify any emerging themes or interests, the sparsity of occurrences made this a difficult task. However, while unable to be definitive in terms of trends, clear commonalities across the documents in this group are identifiable. Conspicuously, 40% of the sources in the Green category reference ethics or principles in their title. Although the CoE collection tracks any AI-related initiatives and is not limited to those with an ethics focus, it is notable that so many sources with an ethical perspective are included in this Green grouping, significantly more than in the other two groupings. Reflective of their origination in international organisations such as UNESCO, many documents here concern the applications of AI in education, health and public sector settings, as well as sharing a focus on interdependence, human-centric AI and a diversity of cultural expressions. A fourth and final thematic connection observable across the Green sub-corpus is the presence of a consideration of the environmental and sustainability implications of AI. This again is different from findings from the Red and Amber sub-corpora.

## Discussion

Our findings presented in the previous section could perhaps more accurately be described as non-findings, in the sense of the significant dearth of reference to underrepresented populations and voices from the Global South. Of the large corpus (*N* = 463) analysed, a third make no reference at all to any of the anchor search terms or associated codes. Furthermore, of the documents in which one or more search terms are present, the overall frequency of occurrences is extremely low, usually just a single reference and not enough to constitute a focus or theme of the given document. Based on a corpus representing the broad AI landscape, embracing national strategies, policy papers and binding instruments and covering a breadth of AI fields, from robotics to blockchain to healthcare to ethical design, the findings from this analysis provide compelling evidence that there is little consideration in the documents of the Global South, the SDGs and underrepresented populations. Suggestive of dominance by the Global North in AI policy and practice, what are the implications of such findings? If the development of AI policies, the practical deployment of AI, as well as the discussion around its ethical frameworks, are being driven by the Global North and Western epistemic traditions, what are the risks for cultural and ethical diversity, global fairness and interdependence?

Before discussing these points in greater depth, it is pertinent to address the issue of geographical bias in the corpus. As previously noted, while the corpus is large and diverse in many ways (initiative type, source), the predominance of three countries (US, UK, Germany) and two agencies (EU, CoE) in particular, is clear. All representing more economically developed countries and a Global North perspective, it could be suggested that the corpus is itself influenced by a language bias for English in the original CoE database, upon which the corpus was developed. However, while the Council acknowledges the data collection by the Secretariat is non-exhaustive, it is certainly comprehensive in its breadth of AI initiatives which encompasses 15 languages and 55 different countries as well as international agencies. As found in other, smaller, AI literature collections and synthesis papers, the proliferation of AI documents is coming from the Northern or Western world [13, 14, 86, 87]. Therefore, this broad corpus can be considered representative of the existing AI literature and the findings from its analysis are generalisable and thus applicable more widely.

**Fig. 3:**
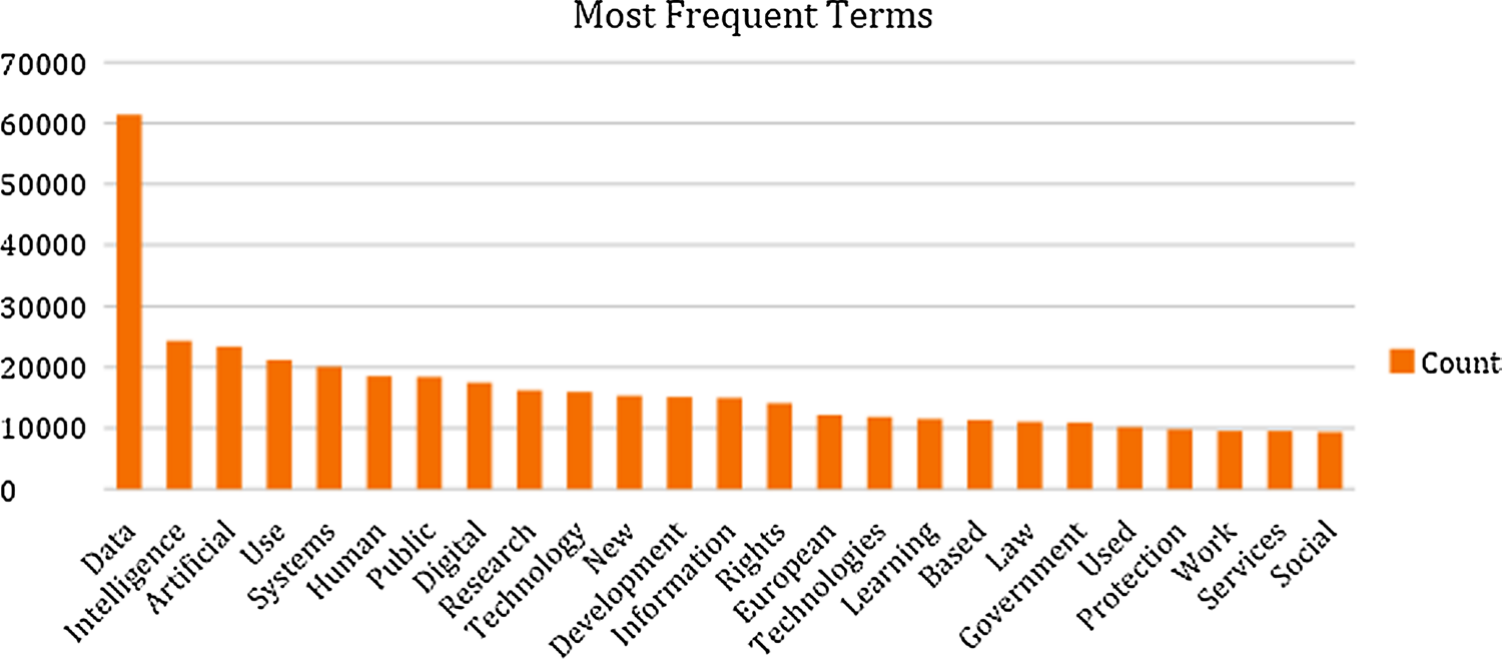
Most frequently occurring terms

At this point, having identified a lacuna associated with underrepresented voices in the literature, it is worth examining what the AI documents within the corpus are concerned with in thematic or conceptual terms. Analysis of the most frequently occurring terms in the corpus (see Fig. 3) reveals a concentration, understandably, on words such as ‘data’, intelligence’, ‘artificial’ ‘technology’ and ‘systems’. While ‘human’ and ‘rights’ feature in the top 25 terms (at sixth and fourteenth, respectively), there is a distinct focus on ‘law’, ‘government’, ‘research’ and ‘development’. Interestingly, ‘European’ is at number 15 in this list of frequently occurring terms, confirming the prevalence of EU and CoE-sourced initiatives. While the most common words could be suggestive of a utilitarian or legal approach to AI, an exploration of the corpus in terms of concepts, as provided on the CoE site, reveals a focus on privacy, human rights, transparency, responsibility, trust and accountability. Notably, sustainability and sustainable development both feature but not to a great level, in keeping with the findings of this research.

The general absence of any meaningful or deep engagement with issues of underrepresentation and inclusion in terms of a lack of attention to diverse socio-cultural and socio-economic contexts, across such a large corpus of AI literature is an important and worrying gap for several reasons. Symptomatic of a lack of diversity of voices in the field, it is challenging that this ever-expanding body of work on AI ignores or is unconcerned with such considerations, given the normative potential and influence of such work. Ultimately, this means the perpetuation of a strongly Global North perspective around AI and its governance, which may not be appropriate or even applicable in any broad manner outside the geographic, social, cultural and ethical milieu in which it was developed. As noted, AI is not confined to one geographic location but is being and will continue to be deployed globally and the absence of real consideration of different contexts and value systems is problematic. This is especially so regarding the impact of AI implementation amongst overlooked groups whose input is not sought and who remain outside any public AI discussion.

Moreover, the results from the analysis of the three sub-corpora in this study are strongly indicative that AI policy development and governance lacks a diversity of voices. Within the Red sub-corpus, where there is not a single reference to any of the search terms or codes, two-thirds (66%) of these documents are AI policy papers or guidelines. As such, it appears that the priorities of the Global South are largely excluded and not of parity with those of the Global North across these AI policy papers. Suggestive of a lack of consideration of the diversity of national and local contexts in what is being put forward as policy and regulation of AI, there is further evidence in the Amber literature. Consisting of documents where search terms occur but only constitute between 1 and 2% of the content, this sub-corpus is dominated by policy papers and reports (70%). This is indicative that AI policy is neither inclusive nor diverse with implications for the global adoption and implementation of such strategies and plans. Appendix 4 presents the breakdown of documents by number in each sub-corpus, according to source or country/agency of origin. Of note in this regard also is the fact that within the Green sub-corpus, which represents the small number of documents where search terms and codes are present, there is not a single item from the UK. Despite being a key contributor to the weight of documents in the overall corpus (the second highest provider country), none of the UK’s AI documents were sufficiently concerned with the theme to be categorised as Green. Of further interest is the finding that within the Green sub-group of literature is a proliferation of documents with explicit reference to ‘ethical’ or ‘ethics’ in their title. As many of these focus on the ethics of AI, it could be a positive indicator that these sources pay some attention to broader issues around inclusion. However, it is also evidence that such literature is a small minority in a large corpus and may not be a priority for policy makers or legislators.

What gets debated and put in the public domain is important to the development of AI policy and governance. Reflective of the dominance of Global North perspectives, ideas and voices, this study demonstrates that there is a narrowness of representation across the AI literature, with a lack of equity in priorities being addressed in the policy documents. The potential impact of this on communities with different values, ideals, social and cultural mores to prevailing individualist or liberal belief systems cannot be overlooked. For example, AI solutions may be developed in the Global North that are incompatible with ethical systems in the Global South and therefore not suitable for these environments. Moreover, a Global North-focussed AI solution may not be able to deliver the promises of various AI4Good missions. Within the Global North, a lack of focus on the issue of underrepresentation can exacerbate possible blind spots within its own communities. AI policy, principles and ethics could and must better represent the diversity of the global community into which AI is being deployed. To help redress this imbalance in representation and thought, suggested approaches are proposed in the final section.

## Conclusions, limitations and future research

Using a corpus built from a database of URLs referenced in the Council of Europe tracker of AI initiatives, this paper assessed an assembly of AI documents (*N* = 463) for reference to 11 anchor terms and more than 60 associated codes relating to the Global South and other underrepresented populations. Representing a significant extension of a previous study of grey literature on AI ethics (*N* = 84) which found blind spots in the soft law documents with regard to gender more broadly and to the Global South, this study encompasses 10 categories of documents from 8 different types of issuer. However, despite the broadened extent of the corpus, the results described and discussed above bear a striking similarity to those of our initial study in the previous iteration of our work [18].

While other research on this topic identify the low contribution of places such as Africa in the literature of AI ethics and the predominance of Global North values and vision around the ethics of AI [46, 55], they are more observational in nature. One of the main contributions of this study is the provision of evidence-based and substantiated corroboration confirming the prevalence of Global North and liberal ethics concepts across the AI literature. We posit that the sample chosen is representative of the body of literature on AI ethics guidelines, polices, frameworks and charters so that any inferences from the sample can be made with confidence. Given the independent sourcing of the sample, by use of an extant corpus, any implicit selection bias on our part has been avoided. In addition, the size of the sample is significantly larger than in previous studies and so can help verify the variety and extent of the corpus selected. The identified absence of voices and ideas from the Global South or other non-Global North perspectives in AI ethics documents here identified, means there is little consideration of the impact of such policies and approaches on other communities. While the study cannot attest to whether this is a deliberate omission or simply oversight, it can conclude that continued underrepresentation will have significant implications for the reinforcement of ethnocentric ideologies and associated power and social structures.

Also of importance is that power asymmetries are often the cause of inequality and understanding the interplay of inequality and power dynamics in AI ethics is essential to prevent the further perpetuation of such inequalities. In order for AI to benefit all, innovative policies and approaches are necessary to govern the ethical use of AI. Just as technological change “*does not occur in a vacuum but is shaped by economic and social processes*” [17, p. 19], so too can policymakers influence the direction of this change in ways that enhance inclusion. For example, a focus on an AI value of efficiency could lead to AI replacing tasks performed by humans, disproportionately affecting those already at risk of poverty in society. However, such outcomes are not inevitable, and AI could just as easily be directed to create new tasks for humans, leading to reduced inequalities [17]. In anticipation of a global deployment of AI technology, a wider, more inclusive engagement with the ethics of AI is necessary: an engagement that accounts for a diversity of socio-cultural contexts and embraces non-Western epistemic thought.

Although this is but one of many areas to be addressed in the ethics of AI, it is an important one as such questions around the role of cultures and contexts in AI ethics are fundamental to developing a robust and inclusive dialogue. Moreover, emerging policies, guidelines and ethical frameworks around AI can take cognisance of the need for a more diverse and intercultural awareness. While the focus on key term search as an analytical tool might be considered a limitation of the methodological approach here adopted, it nonetheless serves its purpose as a quantitative filter on a large corpus. As the body of AI ethics documents continues to expand, a semi-automated quantitative approach is an appropriate one for initial analyses of corpora. Furthermore, it can scale to other languages and more researchers could use this tool and approach to identify other areas of interest in the documents or indeed, other lacunae.

Building on the quantitative analysis, deeper, qualitative evaluation will be undertaken as part of planned further study, with thematic coding to enrich discussion. An alternative approach for analysis is our use of the lens of intersectionality, which identifies the nuanced power structures in play and the multiple ways in which disadvantage is experienced. Although not without its limitations, using intersectionality as a methodology for research which moves beyond its original application to race and gender, can draw attention to and help address policy fragmentation around AI ethics. While AI is good at finding hidden patterns and data is sensitive to areas of intersectionality, intersectional evaluation can help make visible underrepresented identities and unknown biases. Analysis of a single protected attribute does not account for the fullness of intersectional features and may reinforce bias and unfairness. Based on this, our future work will build on alternative ethical stances, using intersectionality to account for social, political, cultural, epistemological and ethical contextualisation of AI ethics, through the application of intersectionality as both critical inquiries and as praxis [76, 88]. We intend to use intersectionality as a data generation tool, through the conduct of qualitative interviews to explore alternative ethical positions, as well as a framework for analysis of that data. It is hoped this work will enable the development of contextualised, reflexive and meaningful understandings of lived experience.

Calling for a more balanced and complete consideration of AI ethics, this study highlights the possibility of achieving this through future research in the further application of decolonial and intersectional theory. From the data analysis of this work, adopting such theoretical approaches could be valuable in addressing the identified lack of diversity. Such work, by situating socioeconomic and geopolitical power in a history of decolonial thought and connecting to the intersectionalities of identity, signposts ways to incorporate the concerns and interests of the Global South, while at the same time acknowledging that the Global South, like the Global North is not a single entity with shared experience or a common epistemic tradition. Incorporating different views, frameworks and epistemologies into the AI ethics discussion is now essential, and can only enrich the ongoing debate and improve the potential of AI technologies to address challenges on a global scale in an appropriate social, cultural, political and ethical manner.

## Appendix 1

See Table

**Table 2:** AI initiative by country or origin of issuer

Country	Number	Final number
Argentina	1	1
Australia	6	5
Austria	5	4
Belgium	5	5
Brazil	2	2
Canada	11	11
Chile	1	1
China	9	9
CoE	48	47
Colombia	1	1
Czech Republic	1	1
Denmark	4	4
Egypt	1	1
Estonia	1	1
EU	62	60
Eurocontrol	1	1
Europol	1	1
Finland	9	8
France	18	17
G20	2	2
G7	1	1
Germany	37	36
Hong Kong	1	1
Hungary	1	1
ICDPPC	1	1
Iceland	1	1
India	2	2
Indonesia	1	1
Ireland	2	2
Israel	1	1
Italy	3	3
IWG DP Telecoms	1	1
Japan	7	7
Kenya	1	1
Latin America	1	1
Lithuania	3	3
Luxembourg	3	3
Malaysia	1	1
Malta	3	3
Mexico	1	1
Multistakeholder	6	6
Netherlands	12	12
New Zealand	1	1
Nordic Cooperation	1	1
Norway	2	2
OECD	9	9
OSCE	1	1
Philippines	1	1
Poland	4	3
Portugal	1	1
Qatar	1	1
Russian Federation	2	2
Saudi Arabia	1	1
Serbia	1	1
Singapore	4	4
Slovakia	4	3
Slovenia	1	1
South Korea	2	2
Spain	5	5
Sweden	7	6
Switzerland	7	7
Taiwan	1	1
Tunisia	1	1
UAE	3	2
UK	43	42
Ukraine	1	1
UN	2	2
UN OHCHR	1	1
UN UNESCO	11	11
UN UNICEF	1	1
UN UNICRI	2	2
USA	71	71
WEF	5	5
	476	463

^2^.

## Appendix 2

See Table

**Table 3:** AI initiative by origin and document type

Origin	Final number
Academia	33
Civil society	24
International organisation	146
Multistakeholder	36
National authorities	143
Private sector	59
Professional association	14
Think tank	8

Type	Final number
Academic paper	8
Binding instrument	5
Meta-analysis	7
Methodology	4
Non-binding instrument	27
Parliamentary proceeding	2
Policy paper	142
Principles/guidelines	130
Report/study	136
Research project	2

^3^.

## Appendix 3

See Table

**Table 4:** Results per search terms

Anchor search term	Results
Sustainable	Present in 231 of 463 (49%)
SDG	Present in 93 (20%)
Global South	Present in 25 of 463 (5.39%)
Low/middle income	Present in 29 of 463 (6.26%)
Developing world	Present in 102 of 463 (22%)
Low resource	Present in 10 of 463 (2%)
Africa	Present in 78 of 463 (17%)
Third world	Present in 3 of 463 (0.6%)
Latin America	Present in 48 documents (10%)
India	Present in 88 documents (19%)
Asia Pacific	Present in 96 documents (21%)

4.

## Appendix 4

See Table

**Table 5:** Results per sub-corpora by number and source

Source	Number
*(1) Red sub-corpus*	
Australia	2
Austria	2
Belgium	2
Canada	4
China	2
CoE	22
EU	12
Finland	1
France	6
Germany	18
Iceland	1
Ireland	1
Israel	1
Japan	1
Lithuania	1
Multistakeholder	2
Netherlands	5
Nordic Co-operation	1
Norway	1
OECD	1
Philippines	1
Poland	3
Russian Federation	1
Singapore	1
Spain	1
Sweden	1
Switzerland	1
Taiwan	1
Tunisia	1
Ukraine	1
United Arab Emirates	2
United Kingdom	14
United States	37
World Economic Forum	1
*(2) Amber sub-corpus*	
Argentina	1
Australia	2
Austria	2
Belgium	3
Brazil	2
Canada	6
Chile	1
China	6
Colombia	1
Council of Europe	25
Czech Republic	1
Denmark	4
Estonia	1
EU	45
EUROCONTROL	1
EUROPOL	1
Finland	6
France	10
G7	1
Germany	14
Hong Kong	1
Hungary	1
ICDPPC	1
India	1
Indonesia	1
International Working Group on Data Protection on Telecommunications	1
Ireland	1
Italy	3
Japan	4
Kingdom of Saudi Arabia	1
Lithuania	2
Luxembourg	2
Malaysia	1
Malta	3
Mexico	1
Multistakeholder	4
Netherlands	6
New Zealand	1
Norway	1
OECD	7
OSCE	1
Portugal	1
Qatar	1
Russian Federation	1
Serbia	1
Singapore	3
Slovakia	2
Slovenia	1
South Korea	2
Spain	4
Sweden	3
Switzerland	6
UN	5
United Kingdom	28
United States	29
World Economic Forum	4
*(3) Green sub-corpus*	
Australia	1
Canada	1
China	1
Egypt	1
EU	3
Finland	1
France	1
G20	2
Germany	4
India	1
Japan	2
Kenya	1
Latin America	1
Luxembourg	1
Netherlands	1
OECD	1
Slovakia	1
Sweden	2
UN	12
United States	5

^5^.

## Footnotes

1 https://manyfesto.ai/.

2 https://www.coe.int/en/web/artificial-intelligence/national-initiatives.

3 https://voyant-tools.org/.
